# A Stochastic Model to Study Rift Valley Fever Persistence with Different Seasonal Patterns of Vector Abundance: New Insights on the Endemicity in the Tropical Island of Mayotte

**DOI:** 10.1371/journal.pone.0130838

**Published:** 2015-07-06

**Authors:** Lisa Cavalerie, Maud V. P. Charron, Pauline Ezanno, Laure Dommergues, Betty Zumbo, Eric Cardinale

**Affiliations:** 1 CRVOI, Centre de Recherche et de Veille sur les maladies émergentes dans l’Océan Indien, F‐97490 Sainte Clotilde, La Réunion, France; 2 CIRAD, UMR CMAEE, F‐97490, Sainte Clotilde, France; 3 INRA, UMR 1309 CMAEE, F‐34398, Montpellier, France; 4 AgroParisTech, F-75005, Paris, France; 5 Université de la Réunion, F‐97715 Saint Denis, La Réunion, France; 6 INRA, Oniris, LUNAM Université, UMR1300 BioEpAR, CS 40706, F‐44307, Nantes, France; 7 GDS Mayotte—Coopérative Agricole des Eleveurs Mahorais, F-97670 Coconi, Mayotte, France; 8 ARS OI, F-97600 Mamoudzou, Mayotte, France; The University of Texas Medical Branch, UNITED STATES

## Abstract

Rift Valley fever (RVF) is a zoonotic vector-borne disease causing abortion storms in cattle and human epidemics in Africa. Our aim was to evaluate RVF persistence in a seasonal and isolated population and to apply it to Mayotte Island (Indian Ocean), where the virus was still silently circulating four years after its last known introduction in 2007. We proposed a stochastic model to estimate RVF persistence over several years and under four seasonal patterns of vector abundance. Firstly, the model predicted a wide range of virus spread patterns, from obligate persistence in a constant or tropical environment (without needing vertical transmission or reintroduction) to frequent extinctions in a drier climate. We then identified for each scenario of seasonality the parameters that most influenced prediction variations. Persistence was sensitive to vector lifespan and biting rate in a tropical climate, and to host viraemia duration and vector lifespan in a drier climate. The first epizootic peak was primarily sensitive to viraemia duration and thus likely to be controlled by vaccination, whereas subsequent peaks were sensitive to vector lifespan and biting rate in a tropical climate, and to host birth rate and viraemia duration in arid climates. Finally, we parameterized the model according to Mayotte known environment. Mosquito captures estimated the abundance of eight potential RVF vectors. Review of RVF competence studies on these species allowed adjusting transmission probabilities per bite. Ruminant serological data since 2004 and three new cross-sectional seroprevalence studies are presented. Transmission rates had to be divided by more than five to best fit observed data. Five years after introduction, RVF persisted in more than 10% of the simulations, even under this scenario of low transmission. Hence, active surveillance must be maintained to better understand the risk related to RVF persistence and to prevent new introductions.

## Introduction

Rift Valley fever (RVF) is a zoonotic and vector-borne disease found in many countries of Africa. It has caused “abortion storms” in livestock in numerous countries including Kenya, Tanzania, South Africa and Mauritania, as well as a series of epidemics (200 000 human cases in Egypt in 1977, 155 deaths in Kenya in 2006 and 19 deaths in Madagascar in 2008–2009) [1–3]. Due to global changes (intensification of global trade and human mobility, climate change, etc.), RVF, like other vector borne diseases, has become a genuine threat in other continents [4]. For instance, RVF was reported in the Arabic peninsula in 2000 [5,6].

Persistence of RVF was unexpected in Mayotte, a 370 km² tropical island with 200 000 inhabitants in the Mozambique Channel. RVF antibodies in cattle were identified for the first time in 2004 serum databank [7]. In 2008, human cases were genetically linked to the 2006–2007 Kenya outbreak [8], and thus recent virus introduction through illegal animal import was suspected. After the virus circulation was observed in Mayotte, a risk assessment procedure evaluated the risk of persistence as “low” [9], given the limited number of ruminants and their dispersal, which would slow down the virus spread, the low vector activity at the time of the assessment and the absence of a wild reservoir,. However, between March 2010 and August 2011, RVF incidence remained high among cattle born in Mayotte (12.9% IC95% = [9.6; 16.2]) and the virus was suspected to have become endemic [10]. Recent field studies in Kenya, Mozambique and Tanzania have shown inter-epidemic activity of RVF as well [11–14]. Reemergence of RVF in the Horn of Africa was attributed to abnormally high rainfall linked to El Niño. This could allow, in particular, *Aedes* eggs infected by vertical transmission to finally emerge [15–17]. However, interepidemic or endemic RVF activity has been poorly studied.

Mathematic modelling is a useful approach for investigating disease dynamics and addressing specific hypotheses in the absence of biological experiments or data [18,19]. A theoretical mathematical model had already shown that RVF could persist at least 10 years in a closed cattle population with two vector populations of constant size, *Culex* and *Aedes*, the latter being able of vertical transmission [20]. Persistence was also possible in a second model including a human compartment, and using low and high sets of RVF transmission parameters, but the authors emphasised the need to test their model against real data. They also underlined that the conclusions on endemic equilibrium and sensitivity analysis were obviously highly dependent on model parameterisation and design [21]. No model was tested against persistence data from a real situation so far. Serological studies conducted in Mayotte may now allow that comparison.

More RVF compartmental models have been developed in the recent years. One of the main differences between those models is the use of different vector population dynamics. Vector population dynamics is complex but strongly influences vector-borne disease dynamics. For instance, overwintering is a burning issue in order to understand Bluetongue or West Nile Virus behaviour in temperate regions [22]. Concerning RVF, the first models were based on vector populations of constant sizes [20,21]. More realistic seasonal vector population dynamics were later introduced to study RVF behaviour in specific areas like California [23], Texas [24], South Africa [25], the Netherlands [26] or Tanzania [27]. Vector population dynamics of *Aedes* and *Culex* were obtained from local vector trapping [23,26] or from more general equations for birth and development rates using local precipitation and temperature data [28,29]. Nevertheless, there are places where RVF vector dynamics and abundance are not well established, hence, theoretical curves have to be used. For instance, Chitnis *et al*. used two sets of parameters alternately to describe a wet and a dry season [30]. They defined the minimum vertical transmission level necessary for persistence over time in this setting. Finally, Chamchod *et al*. compared the impact of a sinusoidal and a step function of vector abundance on RVF dynamics [31]. In this model, RVF introduction produced a first epidemic and could then persist at a low sinusoidal level. New epidemics could be driven only by the introduction of new susceptible animals or by a wet season very favourable to mosquito succeeding years of drought. Still, the impact of various seasonal patterns on the drivers of epidemics and persistence was not clearly addressed.

Before focusing on the specific situation of Mayotte, our aim was first to compare the probability of RVF persistence in different seasonal environments, in an isolated population. A theoretical framework was thus needed [32] combining all the exploitable information from previous models and the knowledge we have on the biological system. We built a stochastic compartmental model for the spread of RVF in a ruminant population. Four seasonal patterns were tested, representing a baseline scenario of constant environment and 3 levels of sinusoidal environment, to study the sensitivity of the virus spread and persistence to type and amplitude of seasonal forcing [33]. For each scenario, we performed a sensitivity analysis on the variance of different outputs describing short and long term dynamics. This will help designing specific studies about significantly influential parameters or targeting those parameters for control in each specific scenario. The model was secondly applied to Mayotte, where persistence has been observed since 2004. In order to better fit the model to the observed situation in the island, three serological surveys in Mayotte were conducted between 2012 and 2013 to complete the existing knowledge on RVF serological status of cattle in Mayotte. New data on vector relative abundance in Mayotte were also collected from 2010 to 2012. We reviewed the relevant literature on the competence of the vectors found in the field. These data allowed for adjusting transmission rates to better reproduce the observed seroprevalence. We finally discussed the relevance and the perspectives of our findings.

## Materials and Methods

### Model description

**Fig 1:**
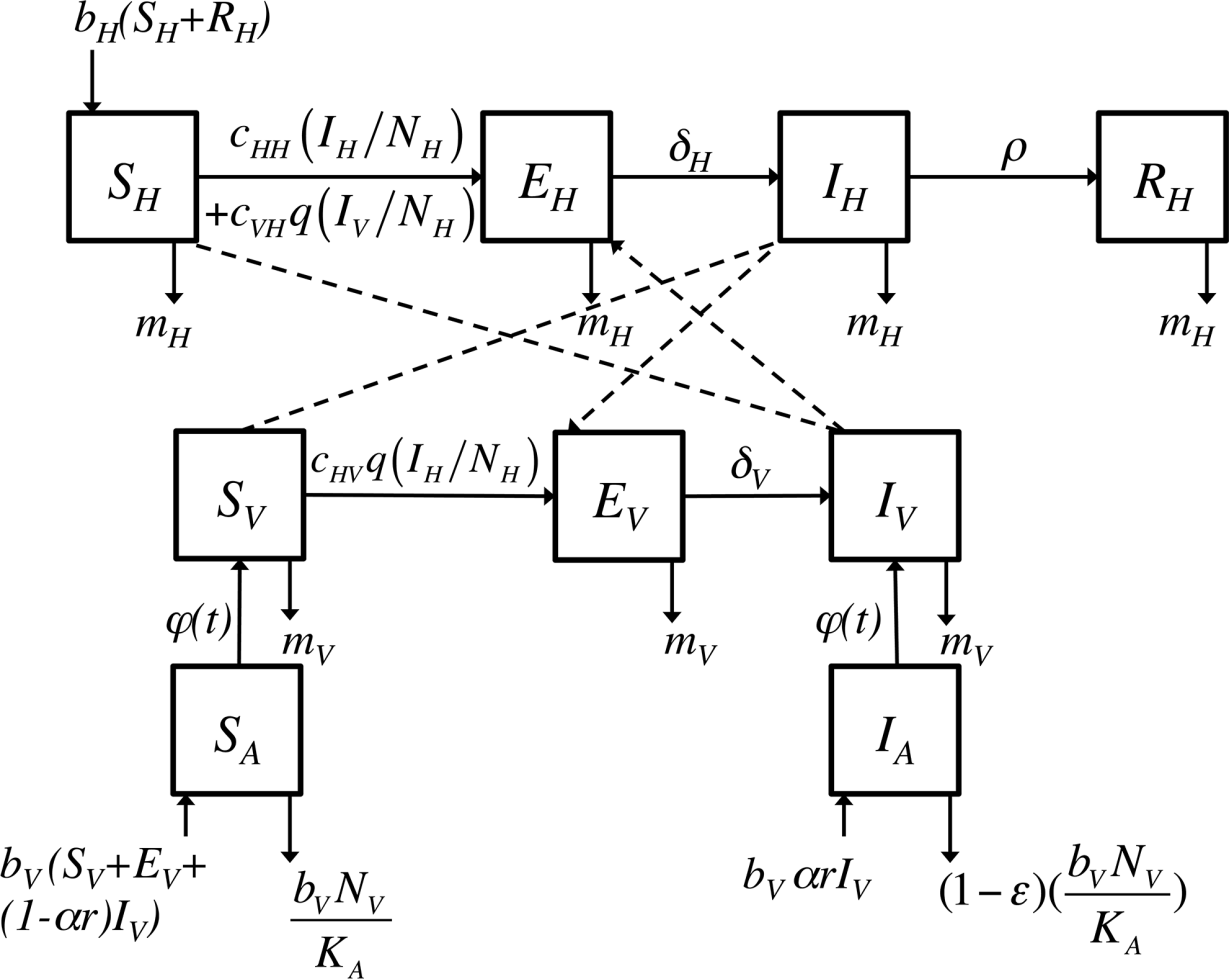
Conceptual model of Rift Valley fever (RVF) transmission. Flow diagram describing the model used for RVF spread in populations of adult mosquitoes (*V*), aquatic stage of mosquitoes (*A*) and ruminants hosts (*H*). Each square represents a health state *X* in population *i* (*X*_*i*_,) with *X = S* standing for susceptible, *E* for latent, *I* for infectious, and *R* for recovered and immune animals. The description, values and references for all parameters can be found in Table 1.

To study RVF persistence, we proposed a stochastic compartmental model (Fig 1) adapted from Gaff *et al*. previous work [20]. Our aim was a better understanding of RVF persistence and humans are unlikely to play a major role [4]. Therefore, we focused on domestic ruminants and vectors but excluded humans. We considered three populations: ruminant hosts (*N*_*H*_), adult vectors (*N*_*V*_) and vectors in the aquatic stage (*N*_*A*_).

Ruminant hosts were divided into four mutually exclusive health states: susceptible (*S*_*H*_), latent (*E*_*H*_), infectious (*I*_*H*_), and recovered and immune (*R*_*H*_) animals. Ruminants could be infected via two routes. First, susceptible ruminants (*S*_*H*_) could be infected by the bite of an infected vector (*I*_*V*_). Infection occurrence depends on the probability of success of the transmission per bite from an infectious vector to a susceptible host (*c*_*VH*_), the biting rate (*q)* and the probability of contact (*I*_*V*_ */N*_*H*_). Second, susceptible ruminants (*S*_*H*_) could be infected by contacts with infectious ruminants (*I*_*H*_) either through direct contacts with biological fluids in case of abortion and slaughter, or through aerosols. The direct transmission depends on the probability of contact (*I*_*H*_ */N*_*H*_) and the transmission rate (*c*_*HH*_). Since this route of transmission is controversial and is assumed to be rare, this transmission rate was very low in our model [34]. After infection, incubating ruminants (*E*_*H*_) became infectious after a latent period of several days (1/*δ*_*H*_) [1]. They remained infectious (*I*_*H*_) for a short period (1/*ρ*), before becoming immunized (*R*_*H*_) for the rest of their life. Birth rate (*b*_*H*_) was assumed to be constant and applied to susceptible and recovered animals. Latent and infectious ruminants were assumed not to give birth, taking into account the very high abortion and neonatal death rate in infected animals [1]. All new-borns were considered to be susceptible (*S*_*H*_). Mortality rate was constant (*m*_*H*_ *= b*_*H*_) and was applied to all health states.

Vectors consisted of *Aedes*, *Culex* and *Eretmapodites* genera all together since data was not available to predict their respective abundance in a general context. The vector population was divided into aquatic (*N*_*A*_) and adult (*N*_*V*_) stages. The aquatic stage (representing eggs, larvae, and pupae all together) was divided into two mutually exclusive health states: susceptible (*S*_*A*_) and infected (*I*_*A*_) individuals. The renewal rate (*b*_*V*_), accounting for the egg laying rate and the survival rate in the different aquatic stages until emergence, was constant and applied to all adult vectors: susceptible (*S*_*V*_), exposed and latent (*E*_*V*_), and infectious (*I*_*V*_) individuals. Individuals in the aquatic stage could be infected (*I*_*A*_) by vertical transmission of RVF which could occur, with a probability *α*, for a proportion *r* of the infected adult vectors (*I*_*V*_) corresponding to *Aedes* [15,34]. Other aquatic individuals were susceptible (*S*_*A*_). Two main processes control the size of the aquatic population: (1) the competition of larvae for food, modelled here with a density-dependent mortality rate with a constant constrain *K*_*A*_; (2) the competition for space at emergence, modelled here with a seasonal function of the emergence rate (*φ(t)*) reflecting the change over time of the carrying capacity for the aquatic stage. The density-dependent mortality rate (*b*_*V*_.*N*_*v*_ */K*_*A*_) did not apply to a proportion *ε* of the infected aquatic stages (*I*_*A*_) corresponding to eggs. We assumed that infected eggs belong only to the *Aedes* genus and therefore are able to resist to desiccation for long periods [2]. The emergence rate (*φ(t)*) allowed vectors to move from the aquatic stage to the adult stage. This rate was assumed to be seasonal, with no prerequisite on the function type, which depends on environmental conditions and the vector species. Such a seasonal pattern has been evidenced recently as a crucial process in mosquito life cycle irrespective of the genus [35].

Adult vectors were divided into three mutually exclusive health states: susceptible (*S*_*V*_), infected and latent (*E*_*V*_), and infectious (*I*_*V*_) individuals. Adult vectors could become infectious via two routes: first, infectious adult vectors (*I*_*V*_) could directly emerge from infected eggs (*I*_*A*_) depending on the emergence rate (*φ(t)*). Second, susceptible adult vectors (*S*_*V*_) could become infected (*E*_*V*_) by biting an infectious host (*I*_*H*_). The force of infection depends on the probability of contact (*I*_*H*_ */N*_*H*_), the probability of transmission per bite (*c*_*HV*_), and the biting rate of adult vectors (*q*). Infected vectors (*E*_*V*_) became infectious (*I*_*V*_) after an extrinsic incubation period (*1/δ*_*V*_) [36]. Adult vectors remained infectious (*I*_*V*_) until they die. The mortality rate of adult vectors (*m*_*V*_), corresponding to one over their lifespan, was independent of their health state. Despite of the existence of studies showing a reduction in the lifespan of mosquitoes infected by arboviruses, this phenomenon has been evidenced and quantified only for *Cx pipiens* in the case of RVF infection [37] and therefore was neglected here.

The model is described by the following system of ordinary differential equations (ODE):

$$\left\{ \begin{array}{l} \frac{dS_{H}}{dt}=b_{H}(S_{H}+R_{H})-\left(c_{HH}\frac{I_{H}}{N_{H}}+c_{VH}q\frac{I_{V}}{N_{H}}\right)S_{H}-m_{H}S_{H}\\ \frac{dE_{H}}{dt}=\left(c_{HH}\frac{I_{H}}{N_{H}}+c_{VH}q\frac{I_{V}}{N_{H}}\right)S_{H}-(\delta_{H}+m_{H})E_{H}\\ \frac{dI_{H}}{dt}=\delta_{H}E_{H}-(\rho +m_{H})I_{H}\\ \frac{dR_{H}}{dt}=\rho I_{H}-m_{H}R_{H}\\ \frac{dS_{A}}{dt}=b_{V}(S_{V}+E_{V}+(1-\alpha r)I_{V})-\left(\varphi (t)+\frac{b_{V}N_{V}}{K_{A}}\right)S_{A}\\ \frac{dI_{A}}{dt}=b_{V}\alpha rI_{V}-\left(\varphi (t)+(1-\varepsilon )\frac{b_{V}N_{V}}{K_{A}}\right)I_{A}\\ \frac{dS_{V}}{dt}=\varphi (t)S_{A}-c_{HV}q\frac{I_{H}}{N_{H}}S_{V}-m_{V}S_{V}\\ \frac{dE_{V}}{dt}=c_{HV}q\frac{I_{H}}{N_{H}}S_{V}-(\delta_{V}+m_{V})E_{V}\\ \frac{dI_{V}}{dt}=\varphi (t)I_{A}+\delta_{V}E_{V}-m_{V}I_{V} \end{array}\right.$$ (Eq 1)

Next, a stochastic counterpart of this ODE system was run in discrete time (using a daily time step) to allow us to estimate the probability of virus persistence. Health transition and mortality rates (depicted by *τ*_*ij*_) were transformed into probabilities (*p*_*ij*_) as follows: for each transition from compartment *i* to compartment *j*, *p*_*ij*_ = 1 –exp(-*∆t τ*_*ij*_). The flow of individuals between compartments *i* and *j* (*∆N*_*ij*_) was then *∆N*_*ij*_ = *Binomial*(*N*_*i*_, *p*_*ij*_), with *N*_*i*_ the number of individuals in compartment *i*. In case of multiple transitions from a given compartment, a multinomial distribution was used [38]. Renewal rate in vectors and births in hosts followed Poisson and binomial distributions, respectively.

### Model parameterization, initial conditions and seasonal emergence scenarios

Parameters used in the model are described in Table 1. The numerical simulations were performed using Scilab 5.3.3 [39] (See S1 Fig for the code). Transmission parameters were set to the averaged low transmission set of Gaff *et al*. [20] for a single vector compartment, taking into account the biting rate.

**Table 1:** Parameters of the Rift Valley fever (RVF) spread model in mosquito and ruminant populations.

**Host parameters**	**Description**	**Value**	**Unit**	**Source**
*b*_*H*_	Birth rate	1/(5x365)	per day	[49]
*m*_*H*_	Mortality rate	*b*_*H*_	per day	-
*c*_*HH*_	Direct transmission rate	1/1000	per day	[1,34,52]
*c*_*VH*_	Transmission probability from vector to host	0.4	-	[20]
*1/δ*_*H*_	Duration of incubation	2	day	[1]
*1/ρ*	Duration of viraemia	6	day	[1]
*NH*_*int*_	Host population size	30 000	animal	[57]
**Vector parameters**	**Description**	**Value**	**Units**	**Source**
*b*_*V*_	Renewal rate	4	per day	[64]
*m*_*V*_	Adult mortality rate	1/20	per day	[20,65]
*q*	Biting rate	1/4	per day	[65]
*c*_*HV*_	Transmission probability from host to vector	0.6	-	[20]
*1/δ*_*V*_	Duration of extrinsic incubation period	6	day	[20]
*r*	Proportion of *Aedes* in the vector population	50%	-	To our best knowledge
*α*	Trans-ovarian transmission probability	1/279 x *r*	-	[15]
*ε*	Proportion of eggs in the aquatic stages	44%	-	[64]
*φ(t)*	Emergence rate	Environment dependent	per day	See *(Eq 2)*
*θ*	Minimum development time before emergence in optimum conditions	5	day	[66]
*K*_*A*_	Carrying capacity	10^6^	mosquito	To our best knowledge

To launch the simulation, a single infectious host was introduced into the ruminant population. Simulations were run over five years.

Vector abundance has been simulated elsewhere either as precipitation and temperature function, available for only few species of RVF competent vectors [24,25,28,29], or parameterized using data collected in the field [23,26]. To assess the importance of different model parameters in the absence of longitudinal data pertaining to the mosquito populations, we simulated four possible scenarios for emergence rates (*φ*_*i*_ *(t)*). Each corresponds to a different rainfall pattern, with the view to study the sensitivity of the model to the type and size of seasonal forcing of vector population abundance. Here is the description of the 4 scenarios that we studied:

- scenario *a*: emergence rate was constant over the year representing a baseline scenario as used in some previous models [20,21,40];
- scenario *b*: emergence rate followed a sinusoidal curve corresponding to a tropical environment where emergence is possible all year-round with a single rainy season. The average value of the emergence rate was equal to the constant emergence rate in scenario *a*. Sinusoidal functions have been widely used to describe vector abundance in the absence of more precise data [22,31,33,41];
- scenario *c*: emergence rate followed here a less favourable sinusoidal curve: same maximal and minimal values as scenario *b* but with a lower mean;
- scenario *d*: the favourable season lasted for half a year during which emergence followed a bell curve pattern with the same maximal values as in scenarios *b* and *c*. In the second half of the year, emergence rate was nil. Adult vectors were forced to die 20 days after the start of the unfavourable period. This scenario corresponds to a more arid environment as described in models applied to East or South Africa [30,42].

Parameter *θ* is the minimal development duration of the aquatic stage. It drives the amplitude of the emergence rate scenarios by setting the maximal value of the emergence rate. In the simulation, the mean development duration was 1/10 days in scenarios *a* and *b*, and 1/16 and 1/15 days in scenarios *c* and *d*, respectively. This covers the observed range of development duration for most mosquitoes which takes from a few days to weeks depending on environmental conditions [20,43,44].

**Fig 2:**
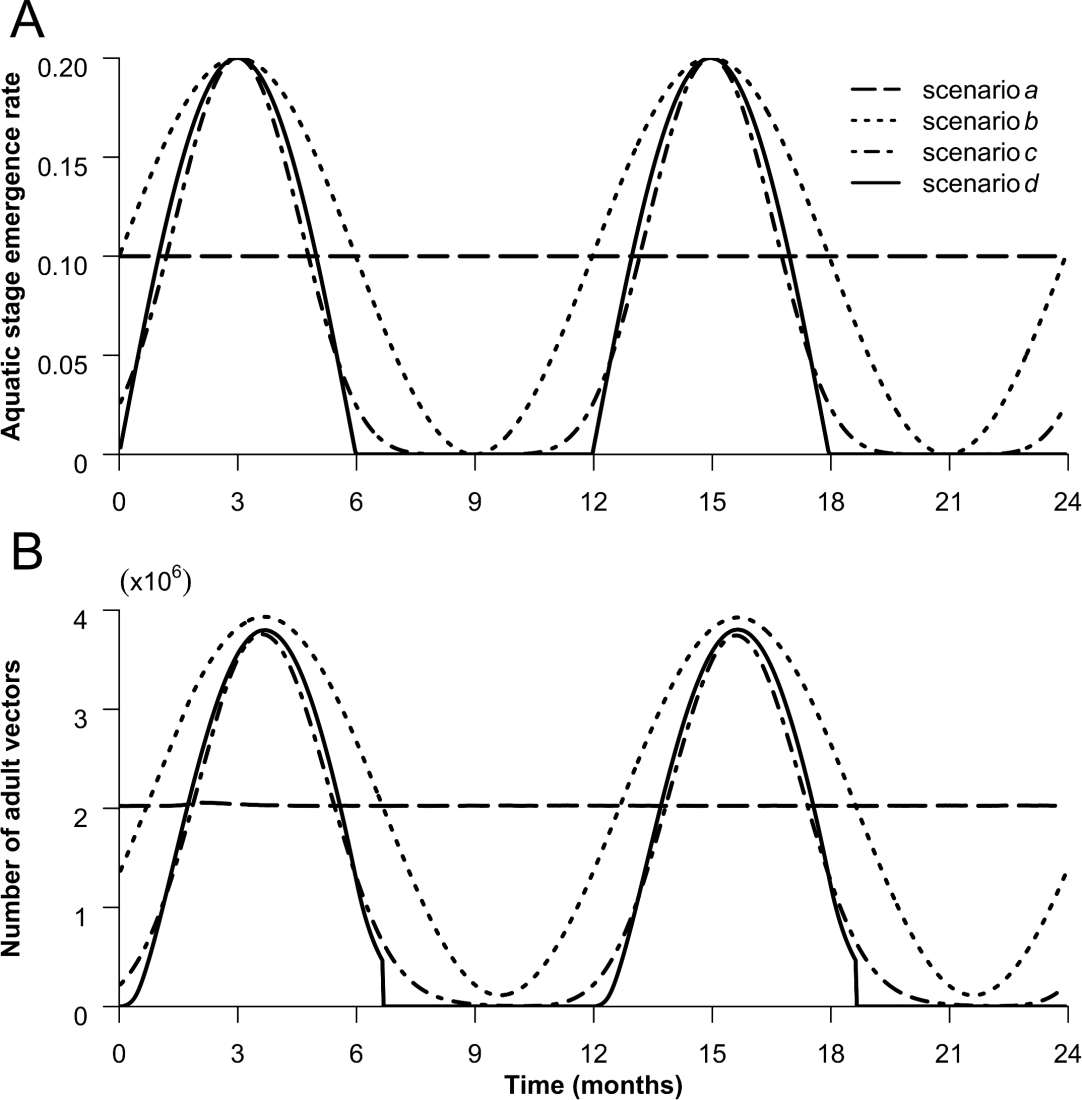
Aquatic stage emergence rate (A) and adult vector population patterns (B). The 4 lines represent the value taken by the emergence rate *φ(t)* and the adult vector population (*N*_*V*_) over time for each seasonality scenario. In the simulations, the one-year pattern is repeated for as many years as needed.

Equations and the resulting population abundance of these four scenarios are described in Eq 2 and Fig 2:

$$\left\{ \begin{array}{l} \varphi_{a}(t)=\frac{1}{2\theta }\\ \varphi_{b}(t)=(\sin(\frac{2\pi }{365}t)+1)\frac{1}{2\theta }\\ \varphi_{c}(t)={\left((\sin(\frac{2\pi }{365}t)+1)\frac{1}{2}\right)}^{3}\frac{1}{\theta }\\ \varphi_{d}(t)=\left\{ \begin{array}{l} \sin(\frac{2\pi }{365}t)\frac{1}{\theta },t\in \left[365(k-1)+1:365(k-1)+182\right],\text{in year}\;k\\ 0,t\in \left[365(k-1)+183:365k\right],\text{in year}\;k \end{array}\right. \end{array}\right.$$ (Eq 2)

### Model outputs

The model predicted the distribution of vectors and hosts in the different health states over time. We also computed the probability of virus persistence over time (hereafter called ‘persistence’), defined as the proportion of the stochastic repetitions in which there was at a given time *t* at least one infected vector (adult or aquatic stage) or one infected host. The number of repetitions was a compromise between steady output distribution and simulation time. Each simulation comprised 1500 repetitions of the stochastic model.

The following aggregated outputs were also considered:

- persistence one and five years after the introduction of the virus into the population;
- mean number of infectious vectors in the aquatic stage (*meanI*_*A*_) in case of persistence, from the second year after introduction until the end of the simulation (excluding the epizootic peak during the first year);
- maximum proportion of infectious ruminants in year one, two, and three, in case of persistence (*maxI*_*Hk*_ in year *k*);
- mean proportion of recovered animals (*i*.*e*. seroprevalence) in case of persistence (*meanR*_*H*_) from the second year until the end of the simulation;
- mean proportion of ruminants infected via direct transmission in case of persistence.

### Sensitivity analysis

First, we explored the model predictions assuming no vertical transmission in vectors for each of the four seasonal scenarios. Second, to evaluate the effect of variations in the different parameters (including vertical transmission) and their first order interactions on the aggregated outputs presented above, we carried out a sensitivity analysis using a Fractional Factorial Design sampling method [45] (using the FACTEX procedure in SAS software. Copyright, SAS Institute Inc. SAS and all other SAS Institute Inc. product or service names are registered trademarks or trademarks of SAS Institute Inc., Cary, NC, USA.) for each of the four scenarios with a variation of 10% below and above the nominal value of each parameter. Following this method, 2187 sets of parameters were computed for each scenario made up of 1500 repetitions. For each output in each scenario, a linear regression model was fitted with all of the principal effects of the parameters and their first-order interactions. The global contribution of parameter *i* (including the principal effect plus interactions in which parameter *i* is involved) to the variation in output *y* was:

$$C_{i}^{y}=\frac{SS_{i}^{y}+\frac{1}{2}\sum {}_{j}SS_{i:j}^{y}}{SS_{tot}^{y}},$$

with the total sum of squares of the model for output y, the sum of squares related to the principal effect for parameter *i* for output y (nil if parameter *i* is not retained in the model), the sum of squares related to the interaction between parameter *i* and parameter *j* for output *y* (nil if this interaction is not retained in the model). The sum of the contributions for output *y* equals the coefficient of determination of the regression model *R* ^*2*^. Statistical analyses were performed with R 2.15.0 [46].

### Case study: the island of Mayotte, France

The model was applied to the island of Mayotte, with vector transmission probability per bite adjusted to what is known about RVF vectors in Mayotte. First, relative abundance of RVF competent vectors was estimated based on recent entomological captures [47]. Reviewing the literature on competence of these species allowed us to gather information on RVF transmission probability per bite for each species. Since transmission is expected to be affected by numerous parameters in Mayotte environment (blood meal in dead-end hosts, lower contact rates due to vegetation and animal handling), an adjusted contact rate coefficient (*k*) was estimated, in this context, by comparing observed seroprevalence found in the literature until our latest seroprevalence survey, in 2012–2013, with the recovered hosts percentage predicted by the model using a range of different set for transmission rates *c*_*VH*_ x *k* and *c*_*HV*_ x *k*. The detailed steps to estimate *c*_*VH*,_ *c*_*HV*_ and *k* are described thereafter.

#### Observed vector abundance and known transmission probability

We considered vector species found in a field study in Mayotte between 2010 and 2012 by the ARS (*Agence Régionale de Santé*, the regional health services) (see [47] for details on the protocol).

Vectors were repeatedly trapped in five farms representing five different ecological sites throughout the island. Dissemination (*DR*) and transmission (*TR*) probabilities for each species were looked for in the literature excluding studies with intrathoracic inoculation of RVF because it could overestimate the dissemination. We averaged the relative abundance for the vector species with an available *DR* (respectively *TR*) over the whole trapping period and over the five sites. Then, the host-to-vector (*c*_*HV*_) (respectively vector-to-host *c*_*VH*_) transmission probability was estimated as the mean of all *DR* values (respectively *TR* values) weighted by the relative abundance of the respective species. Parameter *α* was calculated taking into account the proportion of competent vectors belonging to the *Aedes* genus found in the field study. Mayotte has a tropical climate homogeneous for the whole territory (S2 Fig) with temperature favorable to mosquitoes all year round. The dry and rainy seasons last on average six months each, with rainfall under and above 100 mm per month, respectively (S2 Fig for precipitation and temperature in Mayotte). Natural or anthropogenic sites are available all year round but their abundance depends on the season. Especially in rural areas, *Aedes aegypti* was shown to fluctuate between dry and rainy season [48]. Hence, a sinusoidal seasonality (scenario *b*) was used because it is thought to better fit the entomological dynamics as observed in Mayotte (Thomas Balenghien, personal communication). No difference was made in this model between small ruminants and cattle. Viraemia is indeed about the same for these species [1]. In the absence of precise data on the extensive small ruminant production system in Mayotte (12 619 animals, mainly goats) we used the same value for birth rate as in cattle [49]. Based on our knowledge, there is no significant seasonal variation in ruminant (cattle and small ruminants) birth rate in Mayotte [49]. The day/night cycle is more or less constant and most farmers are not yet willingly trying to control births to adapt them to economic constraints. Hence, we used a constant birth rate.

#### Observed seroprevalence in Mayotte

Seroprevalence data was collected between 2004 and 2011 in the bibliography and three times in the field between 2012 and 2013 in order to compare the model outcome with observations in Mayotte.

**Fig 3:**
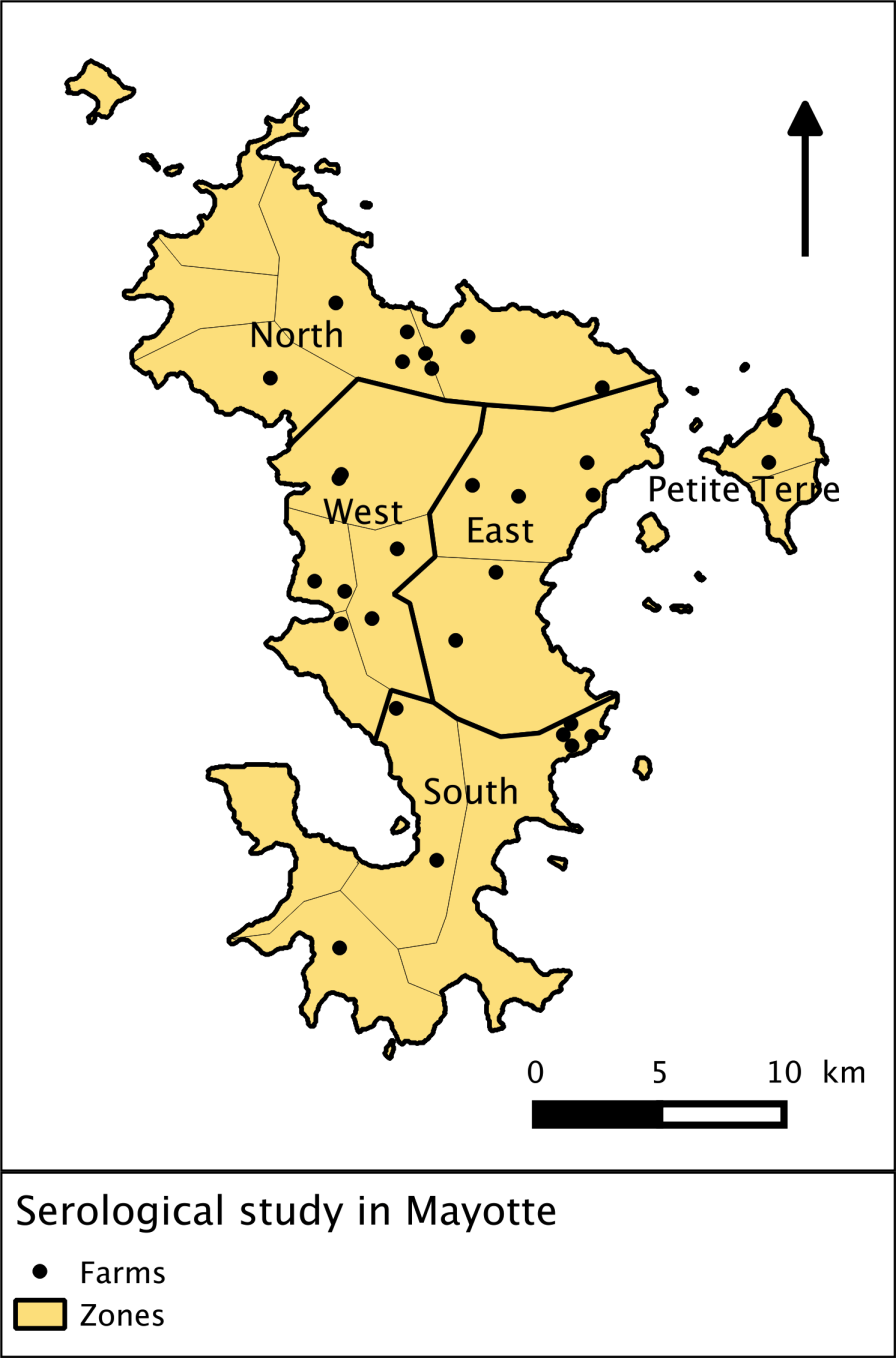
RVF dynamics for different vector emergence scenarios. The 1^st^ column shows the distribution of hosts for each health state: susceptible (*S*_*H*_) in green, infectious (*I*_*H*_) in red and recovered (*R*_*H*_) in blue, for 1500 model repetitions. The 2^nd^ column shows the infectious vectors. The 3^rd^ column shows the probability of virus persistence. Each line corresponds to a scenario (Fig 2).

Search engines were used to find publications on PubMed (http://www.ncbi.nlm.nih.gov/) and ScienceDirect (http://www.sciencedirect.com/) using key words “Rift Valley fever” AND “Mayotte” in English and French. Thirty-two publications were found. Local veterinary services and research institutions were interviewed to find seroprevalence data in unpublished documents, reports or databases. Original datasets were obtained upon request to the authors in order to specify temporal frames and to calculate confidence intervals when necessary.From May 2012 to April 2013, ruminants were selected to obtain a representative sample of the ruminant population of Mayotte. We divided Mayotte into five zones to cover each agro-ecosystem (Fig 3). The 30 farms included in the study were randomly selected from the most comprehensive database available in Mayotte from the *Chambre de l’Agriculture*, *de la Pêche et de l’Aquaculture de Mayotte* (CAPAM, a local public agricultural institution). All ruminants older than six months of age for cattle and three months of age for small ruminants (beyond colostral immunity) belonging to the same owner were eligible for inclusion in the study.

Three rounds of blood sampling were conducted. The first round was collected between May and July 2012, the second between September and December 2012, and the third between February and April 2013. Blood samples were taken from all animals each time, regardless of the results of previous samples. Sera were analyzed in LVAD (Departmental Laboratory of Veterinary Analysis in Mayotte) with ID Screen RVF competition multi-species (IdVet, France), a competitive ELISA kit for the detection of anti-RVF antibodies in ruminant sera or plasma. Seroprevalence and 95% confidence interval taking into account clusterisation of sampling were computed using the “survey” package in R [46,50].

#### Ethics statements for the 2012–2013 serological survey

The study protocol was implemented with the approval of the Direction of Agriculture, Food and Forestry (DAAF) of Mayotte (Project Number NIP42). Animal sampling in this study was not subjected to the approval of ethics committee neither to specific national of international regulations at the time of sample collection. Consent for blood sampling on a herd was obtained from its owner verbally after information in French or Shimaore. The animals were bled without suffering. No endangered or protected species were involved in the survey.

#### Comparison of observed and predicted seroprevalence to estimate the likely contact rate coefficient *k*

The SIR model was finally run with seasonal scenario *b*, adjusted *α*, *c*_*VH*_ and *c*_*HV*_ parameters as explained above. A new parameter was introduced “*k*” to take into account a more realistic contact rate. Transmission rates were then set to “*c*_*VH*_ x *k”* and “*c*_*HV*_ x *k”*. We used the least square approach to estimate *k*. Our aim was to find the value of *k* that minimized the distance between the observed and the predicted seroprevalence. We defined the predicted seroprevalence as the proportion of animals in the recovered status. To compare observed and predicted data, the time of virus introduction in the model was assumed to be the 1^st^ November 2007 when the rainy season started and when approximately the first RVF IgM positive cow was found in Mayotte, in November 2007 [7]. Proportion of hosts already in the recovered status at that date was estimated according to the literature review. Repetitions were kept only if virus persisted more than three years and 7.5 months which was, to date, the minimum known duration for RVF activity persistence in Mayotte [7,10]. For each period of seroprevalence study, the same number of cattle as sampled in the field was sampled in the animal population simulated by the model. For different values of *k* (between zero and one) and for each run, we calculated the sum of square of the differences between the observed and the predicted seroprevalence.

## Results

### Dynamics and persistence for each vector emergence scenario

**Fig 4:**
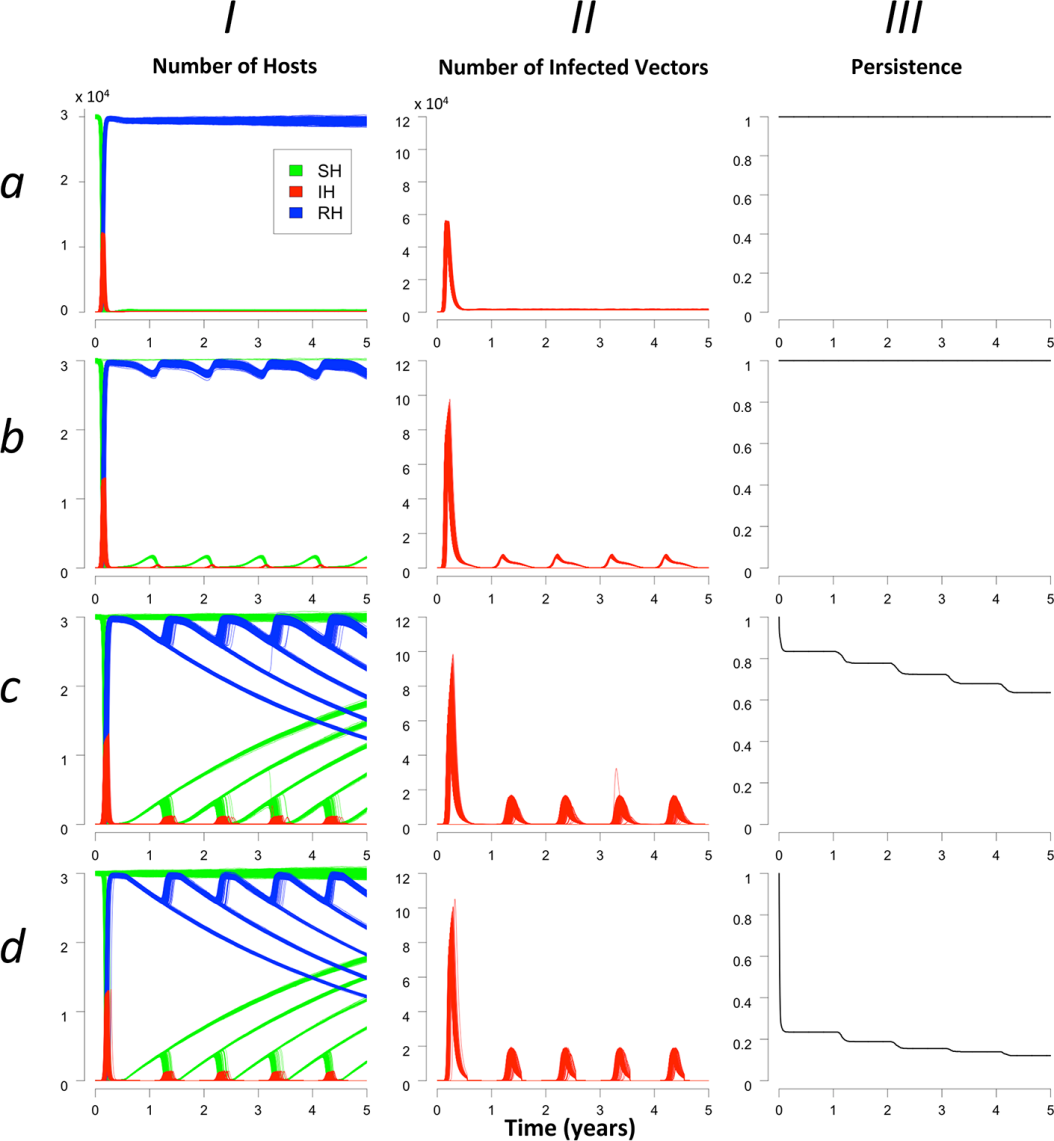
Sampled farms and zones in Mayotte.

The RVF dynamics observed in the simulations are represented in Fig 4. After the introduction of an infectious animal, epizootics could occur in repetitions for all scenarios with a peak of around 40% of the ruminant population being infectious (*maxI*_*H1*_). At the end of the 1^st^ year, in repetitions in which the virus persisted, almost 100% of the host population had become infectious and had recovered. From the second year on, behaviours differed according to vector emergence scenarios. In scenario *a*, new infections occurred constantly with the renewal of the host population so that the infectious hosts remained steady at 0.4% of the population. In the three other scenarios, when the virus persisted, small epizootics occurred early in the favourable season and reached about the same level every year. The maximal proportion of infectious hosts in the 2^nd^ year (*maxI*_*H2*_) attained 1.4, 3.4, and 4% for scenarios *b*, *c*, and *d*, respectively. Peaks always occurred several days (5–15 days on average) before the maximal vector abundance was reached. Yearly maximal proportion of infectious hosts was roughly stable from the 2^nd^ year onwards (*maxI*_*H2*_ and *maxI*_*H3*_). In one repetition (over 1500), in scenario *c*, we observed an increase in year 4 of infectious hosts above the value of previous years, reaching 8% of the host population (Fig 4 section *cI*). Meanwhile, the mean proportion of recovered animals in case of persistence (*meanR*_*H*_) from the 2^nd^ year until the end of the simulation was of 98, 98, 95, and 94% for scenarios *a*, *b*, *c*, and *d*, respectively; the mean number of infected vectors in aquatic stage in case of persistence was very low (1.10^−3^, 2.10^−3^, 5,1.10^−3^, and 8,0.10^−3^% of the vectors in the aquatic stage for scenarios *a*, *b*, *c*, and *d*, respectively). Direct transmission was responsible for very few new cases (5.4 x 10^−2^%, 5.2 x 10^−2^%, 6.1 x 10^−2^%, and 5.7 x 10^−2^% of the total cases for scenario *a*, *b*, *c*, and *d*, respectively).

Without reintroduction, it was possible for RVF virus to persist year after year in the 4 scenarios but with different probabilities. In scenarios *a* and *b*, the virus persisted in 100% of the repetitions over 5 years (Fig 4 sections *aIII* and *bIII*). In scenarios *c* and *d*, the virus persisted respectively in 80% and 20%,of the repetitions after the first year epidemics (Fig 4 sections *cIII* and *dIII*). Extinctions related to a lack of secondary cases occurred during the favourable season and persistence remained stable during the less favourable season. At the end of the 5^th^ year, persistence reached 64% (scenario *c*) and 12% (scenario *d*). When assuming no vertical transmission in vectors, persistence did not change at all for scenarios *a* and *b*. It decreased a little bit faster in scenario *c*, reaching 55% five years after the introduction of the virus. In scenario *d*, RVF virus went extinct without vertical transmission after the beginning of the first unfavourable season, as expected.

### Sensitivity analysis

**Fig 5:**
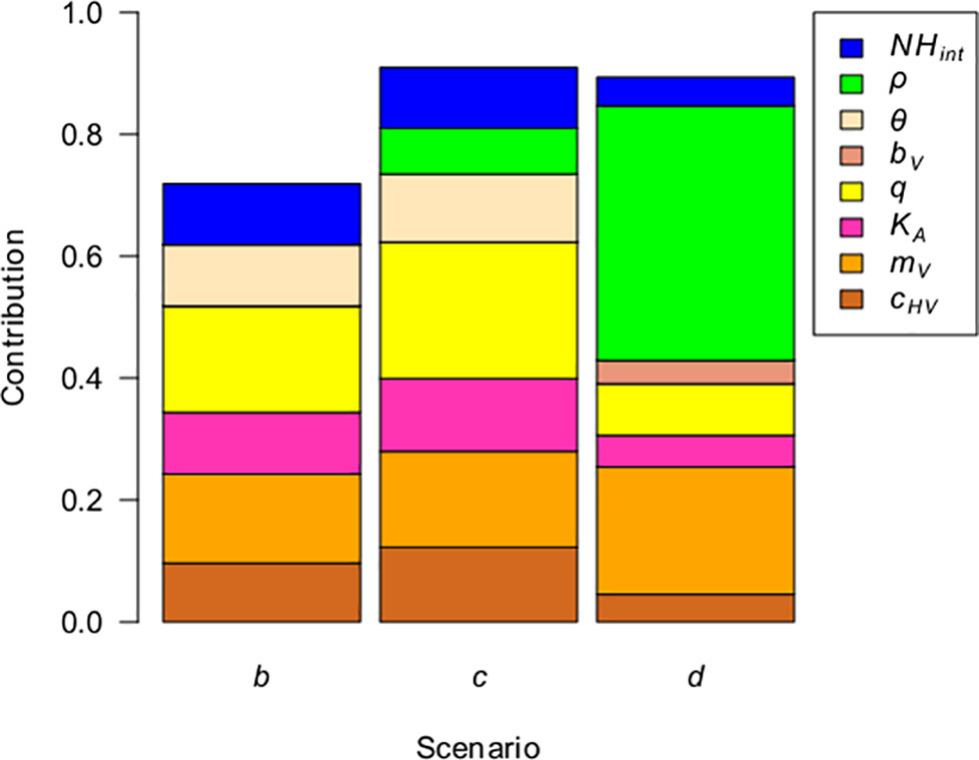
Parameter contributions to the variance of virus persistence one year after introduction. A fractional factorial design was used. Persistence in scenario *a* did not vary and is not shown here. Parameters contributing to more than 2% of the variance have been retained (no interaction contributed to more than 2% of the variance). See Table 1 for parameters, Fig 2 for scenario descriptions and S3 Fig for detailed parameter contributions.

In the sensitivity analysis, in scenario *a*, persistence reached 100% in most simulations. Hence, the contribution of different parameters to the variation of this output could not be analysed. For the other scenarios, eight parameters contributed to more than 2% of the variance of the persistence one year after the introduction of the virus (Fig 5 and S3 Fig) and explained more than 70% of the variance. Parameters common to the three scenarios were host population size (*NH*_*int*_), biting rate (*q*), mortality rate of adult vectors (*m*_*V*_), carrying capacity of vectors in aquatic stage (*K*_*A*_) and transmission probability from hosts to vectors (*c*_*HV*_). They had a similar contribution except biting rate (*q*), which contributed twice more in scenarios *b* and *c* than in scenario *d*. Conversely, viraemia duration (1/*ρ*) contributed to almost half of the persistence variance (43%) in scenario *d*, much less (7%) in scenario *c*, and was negligible in scenario *b*. Parameter *θ*, controlling maximal emergence rate, was influential only in scenarios *b* and *c*. Renewal rate of vectors was influential only in scenario *d* and at a low level (4% of the variance). Persistence five years after the introduction of the virus depended, in scenario *b*, on exactly the same parameters as in the first year. It was impossible to conclude for scenario *c* as the output distribution was bimodal (near 0 or 100%) instead of normal, which is obligatory for analysis of variance. In scenario *d*, biting rate (*q*) and host viraemia duration (1/*ρ*) were the most influential parameters on persistence variance until year five (17% and 24% respectively).

**Fig 6:**
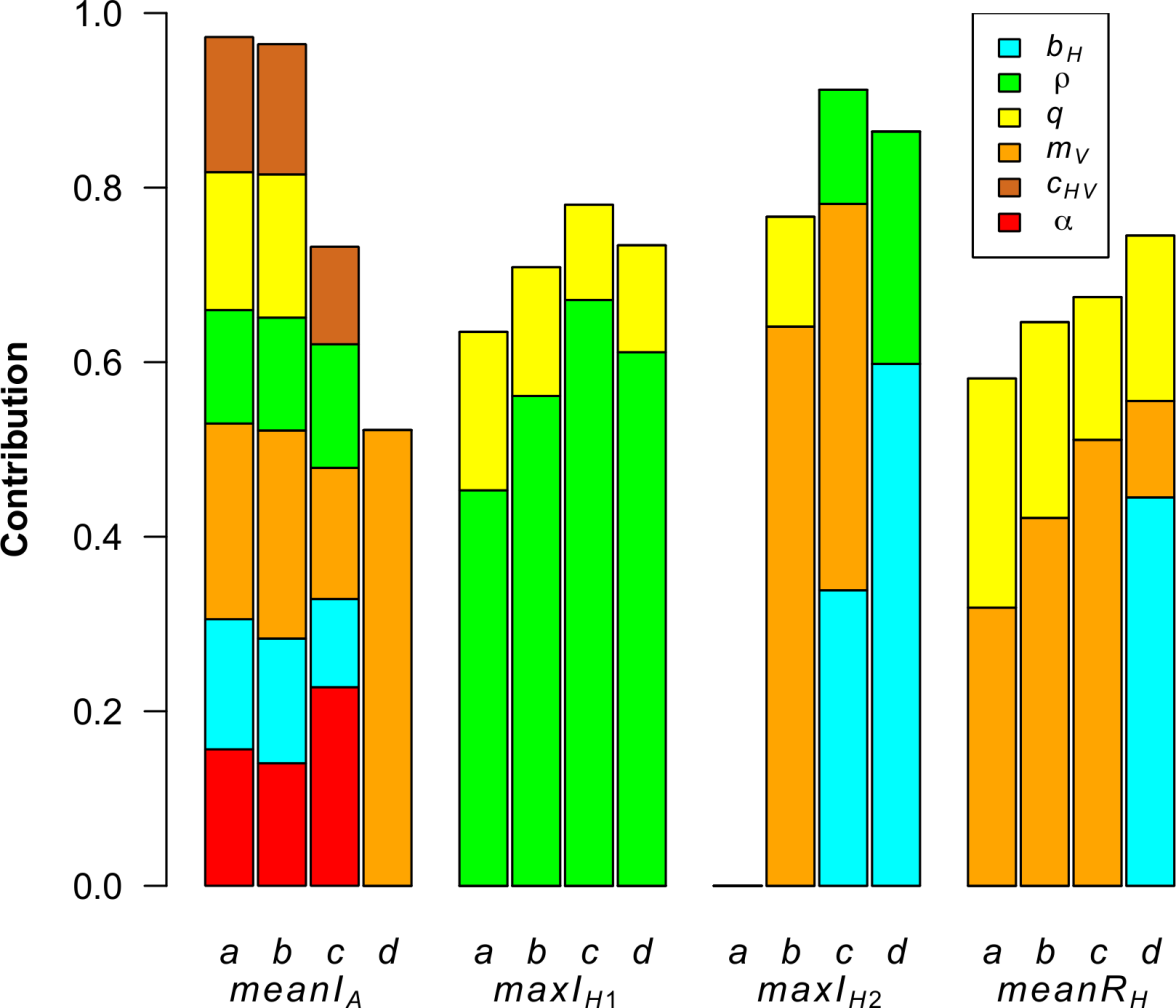
Parameter contributions to the variance of other model outputs. A fractional factorial design was used. To facilitate the interpretation, parameters contributing to more than 10% of the variance are shown. Model outputs are: the mean number of infected vectors in the aquatic stage in case of persistence (*meanI*_*A*_); the maximum proportion of infectious ruminants during the 1^st^ and 2^nd^ years in case of persistence (*maxI*_*H1*_ *vs*. *maxI*_*H2*_); the mean proportion of recovered animals in case of persistence (*meanR*_*H*_) from the 2^nd^ year until the end of the simulation. See Table 1 for parameters and Fig 2 for scenario descriptions and S3 Fig for detailed parameter contributions.

The contribution of parameter variations to the variance of other outputs is shown in Fig 6 and S3 Fig. In all scenarios, adult vector mortality (*m*_*V*_) was the main contributor to variance of the mean number of infected vectors in the aquatic stage (*meanI*_*A*_), which varied from 50 (scenario *a*) to 500 (scenario *d*). For this output only vertical transmission rate (*α*) was shown to be an influential parameter (>2%).

More than half of the variance of the maximal proportion of infectious hosts in the first year (*maxI*_*H1*_) was explained in the four scenarios by viraemia duration (1/*ρ*) and biting rate (*q*). In subsequent years (*maxI*_*H2*_ and *maxI*_*H3*_), the situation differed between scenarios: viraemia duration (1/*ρ*) remained important only in scenarios *c* and *d*, host birth rate (*b*_*H*_) had growing influence in scenarios *b*, *c* and *d*, and adult vector mortality (*m*_*V*_) was influential in scenarios *b* and *c* only.

The variance of the mean proportion of recovered animals in case of virus persistence from year two to the end of the simulation (*meanR*_*H*_) was explained mainly by the adult vector mortality (*m*_*V*_) and the biting rate (*q*) in the scenarios *a*, *b* and *c* and by the host birth rate (*b*_*H*_) and the biting rate (*q*) in scenario *d*.

### Case study: Mayotte

The entomological study conducted by ARS [47] found at least eight mosquito species in Mayotte known to be competent for RVF in the literature (Table 2). 7.84% of the mosquitoes from competent species belonged to *Aedes* genus (*r*). *Cx quinquevittatus* accounted for 45% of the overall diversity but transmission data were lacking for this species. The averaged dissemination and transmission rates were 11.04 and 9.47% respectively.

**Table 2:** Relative abundance and competence studies review of potential RVF vectors found in Mayotte. Diversity was estimated from mosquito collections carried out over two years (2010–2012) in farms in Mayotte, see [47] for protocol details. “NA(>0)” means that evidences were found in the bibliography that the species could disseminate or transmit RVF but no quantified data was available.

Vector species present in Mayotte	Relative abundance (%)	Dissemination rate *DR* (%)	Transmission rate *TR* (%)	Sources
Ae aegypti*	2.38	49.75	3.50	[67–69]
Ae albopictus*	2.43	22.26	5.33	[69,70]
Ae circumluteolus*	0.30	28.00	19.43	[68,71–73]
Ae fowleri*	0.06	40.29	52.00	[74–76]
An gambiae*	1.75	0.75	NA	[69]
Cx antennatus*	2.60	16.50	38.17	[68,77,78]
Cx quinquefasciatus*	45.06	8.33	NA (>0)	[36,67–69,79,80]
Er quinquevittatus*	11.38	NA (>0)	4.55	[67,71]
Others (not known as RVF competent)	34.04	-	-	
	**Total = 100**	***c***_***HV***_ **= 11.04**	***c***_***VH***_ **= 9.47**	

*RVF competent species

**Fig 7:**
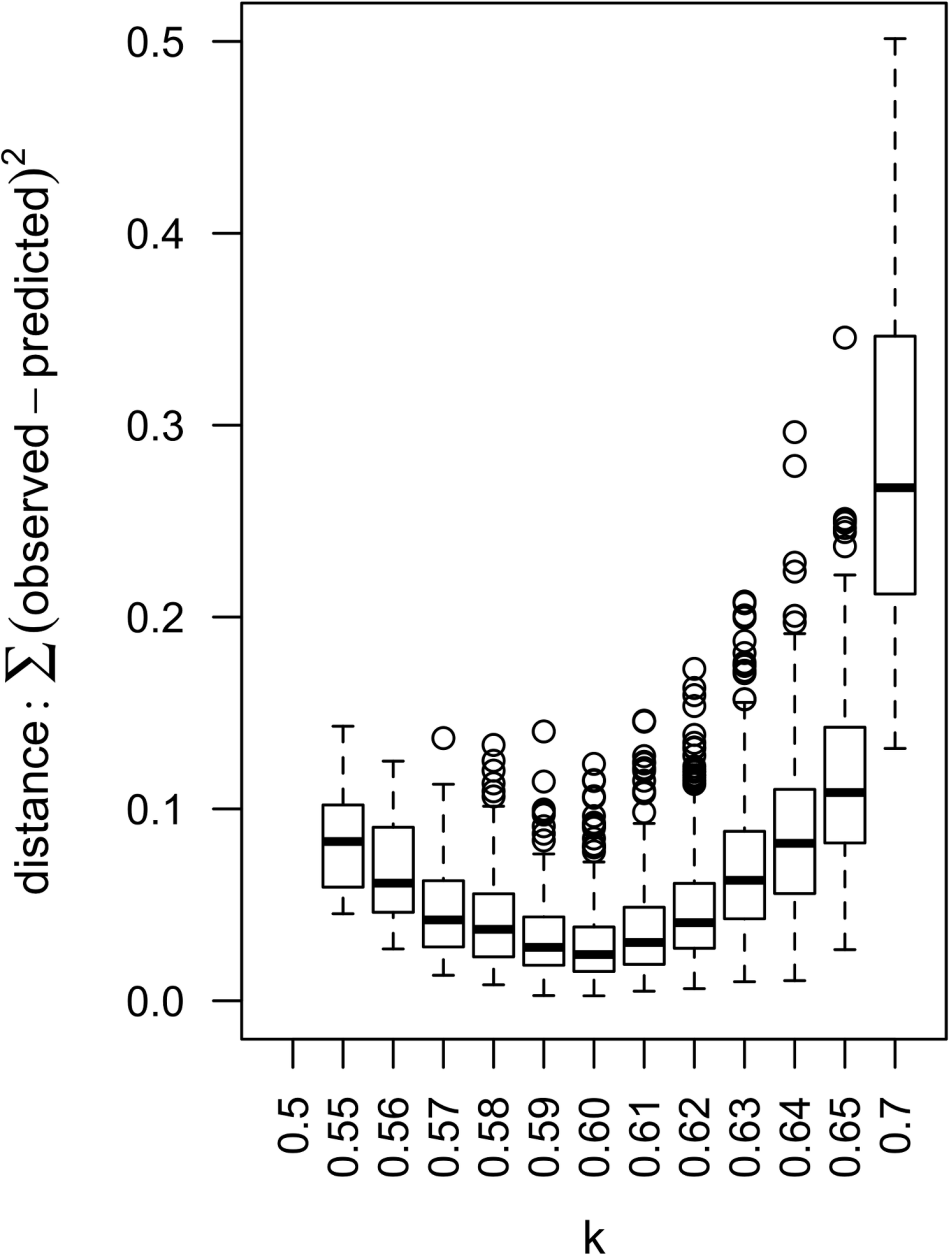
Predicted persistence and host infection dynamics with low transmission rates and observed seroprevalence in Mayotte. (A) Persistence predicted by the model with parameters found in Table 2 and a lower set of transmission rates (*c*_*HV*_ = 0.09, *c*_*VH*_ = 0.04, *k* = 0.6). (B) Host infection dynamics predicted by the model with parameters found in Table 2 and a lower set of transmission rates (*c*_*HV*_ = 0.09, *c*_*VH*_ = 0.04, *k* = 0.6). Susceptible hosts (*S*_*H*_) are in green, infectious (*I*_*H*_) in red and recovered (*R*_*H*_) in blue. (C) Observed seroprevalence in ruminants in Mayotte from 2004 to 2013 is in purple. Seroprevalence predicted by the model, from 2007 on, is in blue. Blue dots represent the median and arrows the 5 and 95% percentiles of the 1500 repetitions.

In the serological survey of 2012–2013, we found 30, 33, and 29 positive animals out of 131, 157, and 161, respectively. Using all available knowledge (literature [7,10], an internal report of 2009 and the 2012–2013 serological survey), the seroprevalence observed between 2004 and 2013 ranged from 3% to 35%. At virus introduction, remaining seroprevalence was set to 16.2% as found in the raw data of the study from Cêtre [7] (Fig 7C and Table 3).

**Table 3:** RVF serology studies in ruminants in Mayotte from 2004 to 2013. Data from 2004 to 2011 were obtained from a literature review. Original data sets could be obtained when source is marked with an asterisk (*). Data from 2012 to 2013 were obtained through new serological surveys. Design effect refers to the clustering effect of the sampling design [81].

Study beginning-end	Number of animals tested	Number of farms	Number *or %* of positive IgG tests	Species	Design effect	Source
Oct 2004 - Feb 2005	243	67	33	Cattle	1.41	[7] *
June 2006—Sept 2006	130	39	16	Cattle	0.84	[7] *
June 2007-March 2008	419	124	68	Cattle	2.39	[7] *
June 2008-Aug 2008	267	12	42	Goat	5.43	[7] *
May 2009-Aug 2009	382	36	135	Cattle	3.46	Dr Sébastien Girard unpublished*
July 2011—Aug 2011	452	33	*25*.*3%*	Cattle, goat, sheep	-	[10]
May 2012-July 2012	131	29	30	Cattle	1.21	- *
Sept 2012—Dec 2012	157	28	33	Cattle, goat	1.68	- *
Feb 2013-April 2013	161	29	29	Cattle, goat	1.52	- *

**Fig 8:**
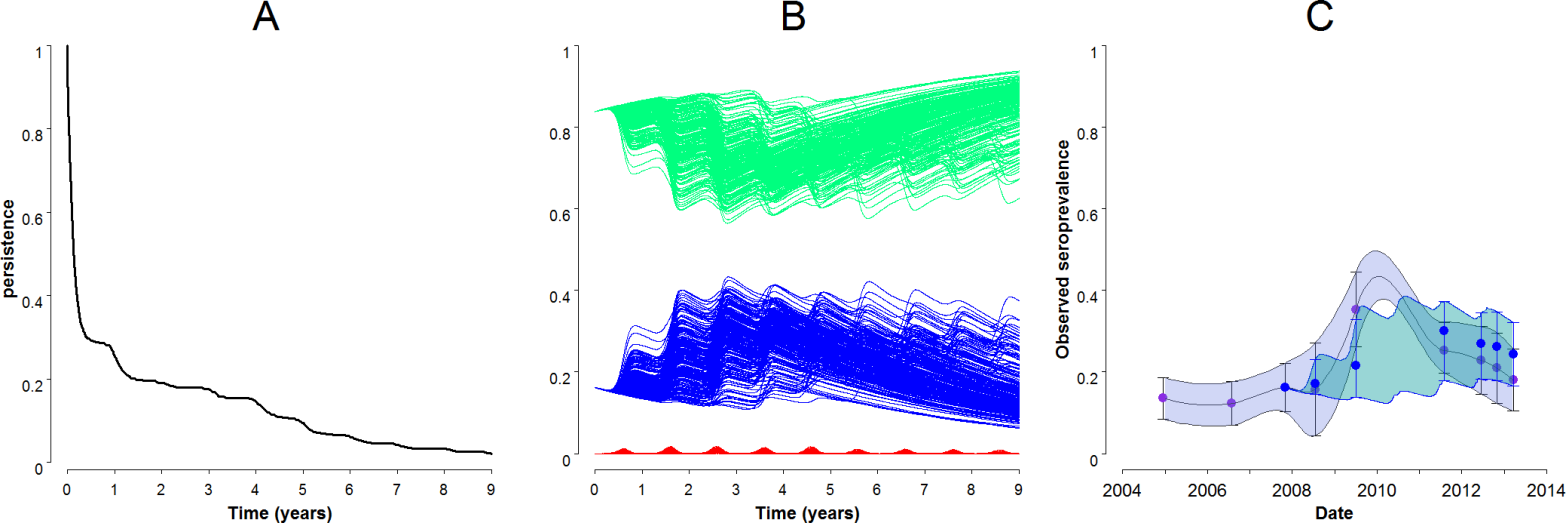
Distance between observed and predicted RVF seroprevalence in Mayotte used to determine the most likely value for transmission rate. Sum of square of differences between observed and predicted seroprevalence were computed for each of the 1500 repetitions for different values of the possible transmission rate occurring in Mayotte (*k*).

Given the model settings, the value of *k* minimizing the distance between observed and predicted data was to be found around 0.6 (Fig 8).

Model predictions with values for *α*, *c*_*VH*_ and *c*_*HV*_ estimated using the entomological study and *k* = 0.6 are found in Fig 8. The virus persisted in 25.5%, 9.5% and 2.1% of the repetitions after one, five and nine years respectively (Fig 7A). Unlike the above four scenarios, the epizootic maximum could be reached in year one, two or three and the maximum proportion of infectious ruminants (*maxI*_*Hk*_) could vary from year to year and reincrease even after few years (Fig 7B). When the virus persisted, the proportion of recovered animals oscillated between 6.2 and 43.3% and the proportion of infectious animals increased at the middle of each year. The predicted seroprevalence varied within the range of observed seroprevalence (Fig 7C). The mean predicted incidence reached 1500, 3000 and 100 new cases per year in the first, second and ninth year after virus introduction, respectively.

## Discussion

This stochastic model is the first one to be used to study RVF persistence probability and its driving parameters over several years under different seasonal patterns of vector abundance. It was able to mimic a large range of virus spread patterns, from obligate persistence in a constant or tropical environment (without needing vertical transmission or reintroduction) to frequent extinctions in an arid climate. In each seasonality scenario, key parameters influencing the model predictions were identified. In addition, the model was applied to a real geographical area (Mayotte) for which we have shown that lower transmission rates than assumed may explain the observed seroprevalence for the last decade. RVF persistence was possible under such a scenario of low transmission for more than 10 years.

### Accounting for vector abundance seasonality in RVF modelling

Accounting for seasonality in vector abundance is a fundamental concern in vector- borne diseases since vector and pathogen biology and development depend on temperature or precipitation. A recent literature review of mathematical models of mosquito-borne pathogens [51] noted that only 14 per cent of them included a variation of vector abundance, either sinusoidally or based on a pattern derived from data. But applying it to Mayotte, with no longitudinal data allowing deriving such equations, is a real challenge. Moreover, while in the Netherlands and California, models could focus on two competent species only, 40 species of mosquitoes are known to transmit RVF virus. In Mayotte, we have found eight species able to transmit RVF virus. The preliminary use of equations from Gong *et al*. [28] on West Nile virus vectors in New Jersey lead to almost constant development and egg laying rates and did not capture the expert knowledge and the tropical seasonality observed in Mayotte [48]. We decided to focus on precipitation pattern influencing vector population dynamics by regulating larval habitats availability [44] in an intertropical geographical area. Average temperatures oscillate between 25 and 28°C all year round in Mayotte and thus cannot impact transmission nor development time significantly as it would do in California or the Netherlands [23,26]. Our conceptual framework allowed us to compare 4 qualitative scenarios.

### RVF dynamics and persistence depending on vector abundance scenarios

In all the four scenarios, RVF persistence five years after virus introduction was possible without the need for neither reintroduction of the virus nor for a wild reservoir. But the probability of persistence varied between scenarios: it decreased as seasonality became less favorable to vectors. This confirms the low level persistence observed in one previous model with constant abundance [20]. In Gaff *et al*. model, epizootic cycles were observed linked with the longevity of the hosts. In our case, despite the low transmission rates (equivalent to Gaff *et al*. low scenario), infection was facilitated by a higher vector/host ratio linked to our tropical environment-oriented model. Our average vector/host ratio is 30 times higher than in Gaff *et al*. model, but only three times higher than in rainy season of Chitnis *et al*. [30] and three times lower than the sparsely populated area of Fischer *et al*. work [26]. In the constant scenario, as soon as new susceptible hosts were born, they were exposed almost immediately to the virus, independently of host population renewal rate. This difference is also noticeable in the size of the first year epidemic peak: around 40% in our model against 0.12% to 12% in Gaff *et al*. work [20]. The important impact of vector/host ratio on epidemic size was also underlined in the recent study of Chamchod *et al*. [31]

The two first scenarios *a* and *b* with same mean emergence rate behaved identically in terms of persistence. Unexpectedly, the two intermediate sinusoidal scenarios *b* and *c* produced very different persistence probabilities confirming the complex influence of type and amplitude of seasonal forcing [33]. A variation in the dry season length, as between scenario b and c, can be misleading about the assessment of the potential of persistence of RVF. Here we showed that, in a tropical humid environment, persistence after one year was at least five times more likely than in the drier environment proposed in scenario *d*.

Subsequent epidemics seem extremely rare (only one run on 1500 in scenario *c*) and would indeed probably require inter-annual precipitation variations, recruitment of susceptible animals or an asynchronous metapopulation system [30,32,52].

### Key parameters controlling RVF dynamics and persistence: use in control measures design

According to this model, persistence control should not rely on the same strategies as epizootic control. To prevent or reduce the impact of the first epizootic, a very quick and efficient vaccination could be beneficial for all scenarios to reduce the duration of viraemia, responsible of 50% of the variation of this output. This supports the strategy suggested by Chamchod *et al*. in a constant environment [31]. For greater efficiency, Gaff *et al*. added the culling to the vaccination [40].

Sensitivity analysis of variance of persistence showed a more complex interplay of parameters than the analysis of first year epidemic peak, but still no interaction was significant. Persistence depended on different parameters for each scenario: it was mainly influenced by the biological traits of vectors in the tropical wet scenarios *b* and *c* and by host related parameters (mainly viraemia) in scenario *d* when a dry season occurred with no vector activity. While persistence behaved the same, at first sight, in the constant and sinusoidal scenarios, their sensitivity analysis diverged. A 10% variation had indeed almost no impact on scenario *a* persistence. In scenario *d*, viraemia controlled the proportion of infected eggs in overwintering eggs; hence influenced the size of the emerging infectious vector population during the next rainy season, and consequently likelihood of RVF persistence. This mechanism of persistence was also suggested for overwintering by previous work [22]. Moreover, for persistence on a longer term, host birth rate became influential by allowing a larger susceptible population to be exposed to minor new epizootics when the favourable season resumes.

It suggests that vaccination would be also useful to prevent persistence in arid climates (scenario *d*). In the other cases, potential targets for control measures include vector mortality rate and biting rate. However, mosquito control is hard to implement in tropical islands such as Mayotte, where animals are spread over large areas of forest in low concentrations. To limit the risk for humans, mosquito control could be employed around houses, although the cost would be high to be truly efficient since part of the population lives in poor construction suburbs or in slums surrounded by dense vegetation and are not accessible to vehicles. Still, transmission mainly via animal slaughtering and maybe via consumption of raw milk (a common traditional practices in Mayotte [53]) would remain possible [1,2,34]. In the future, our model could be used to develop and evaluate control strategies using adapted indicators for seasonal environments, as proposed in Charron *et al*. [54].

Transmission rates barely influenced persistence in this sensitivity analysis design, in accordance to Chitnis *et al*. and Fischer *et al*. conclusions [26,30]. Therefore, uncertainty of their values, in the 10% proposed range above and under nominal values, would barely impact model predictions. Transmission rates were influential only in Gaff *et al*.’s model [20,26,30] which had a much lower vector/host ratio and a wide range of uncertainty. Direct transmission was not a remarkable influential parameter either. Moreover it was unsufficient to allow for persistence in scenario *d*.

Limited information is available on vertical transmission [15]. With a parameter value lower than in earlier models [20,30], and within the range found in other models [26,23], we still observed persistence in our model. The sensitivity analysis showed that infected eggs played a role only in scenario *d* (5% and 13% of variance for *α* and *ε*), confirming previous findings [30]. Vertical transmission in vectors was required in scenario *d* only for overwintering to occur. In the three other scenarios, even without vertical transmission, persistence could occur.

Faran et al. [37] found an impact of RVF infection on the survival of *Culex pipiens*, which is absent in Mayotte. As no further information was available on vectors found in Mayotte, we did not take into account this possible reduction of vector lifespan due to infection. However, this parameter was found as critical in the sensitivity analysis. Therefore, further lab work should be conducted to better estimate this phenomenon. Birth rate is influential in epidemics size and seroprevalence rate, thus further investigations on ruminant life traits in Mayotte (cattle and small ruminant birth rate and pulse) and their evolution with modernization of farming practices are recommended.

### Persistence and transmission in Mayotte

Our work supports the hypothesis, developed in a previous study [10], that RVF has become endemic in Mayotte. It circulated actively on the island with susceptible animals and a favorable environment for mosquito vectors to maintain virus transmission locally [10]. Firstly, field data showed a stability of seroprevalence over the last decade and, secondly, our model could reproduce an endemic situation without an epizootic peak.

We found eight RVF potential competent vector species in Mayotte. *Culex quinquefasciatus* abundance was remarkably high in our trapping compared to a recent study [55], showing the sensitivity of trapping method to their environment (in our case five ruminant farms).

In order to reproduce observed data, transmission rates were divided by more than five compared to other models [20,24,25]. Owing to stochasticity, seroprevalence appeared to be much more variable in this very low transmission situation than in the general framework developed at first. There are several possible explanations for these low transmission rates in Mayotte (1) transmission rates measured *in vitro* cannot be easily extrapolated to field conditions, (2) vector competence of Mayotte vectors has not been measured and can be poor, (3) a recent study [56] suggested that the RVF virus strain circulating in the Comoros in 2011 was hard to detect maybe because of a specific deleted virus strain or a low virus load in animals, (4) the presence of dead-end hosts could lower the biting rate, (5) vector/host ratio might be reduced due to dense vegetation and the relative isolation of herds (mean size of herd: five bovines [57]). The use of low transmission rates may converge with Xue *et al*. model [24] which found that when only few infectious mosquitoes were present at the beginning of the simulation, and thus a low contact rate, with infected ones, the epizootic was longer and the daily incidence was lower. This slower transmission could explain the absence of impact noticeable in the ruminants of Mayotte because no abortion storm in a short period of time would happen.

Interannual climatic variation in vector abundance could have been also added to eventually better explain the seroprevalence peak observed, which the model tends to underestimate. Indeed, above normal rainfall (1996–2010 data obtained from MeteoFrance) observed in 2007-2008-2009 rainy seasons in Mayotte and 2007–2008 climate anomalies underlined by FAO [58] could have facilitated the virus circulation.

A wildlife reservoir, namely buffaloes, seemed to explain persistence in Kruger National Park [42]. It was not considered in Mayotte, since there are no wild ruminants and since other wild mammals (rats and lemurs) have been tested negative for RVF, so far.

Livestock movements may play an important role in the spread of RVF. In Mayotte, there is neither market to gather animals nor transhumance as observed in Eastern Africa. This hypothesis should be further investigated by evaluating more closely movement practices, particularly when the French national identification database (BDNI) will be functional in Mayotte. Nevertheless, mechanisms such as asynchrony of climate between different zones connected by animal movements, as described in Favier et al. [32], were not necessary to allow for persistence here. The maintenance through illegal animal introduction only, exclusively from Anjouan, 70km distant-island of the Union of the Comoros is unlikely. Firstly, RVF circulation level is also very low there (RVF was unnoticeable from July 2010 to august 2011) [59]. Secondly, the illegal transport of ruminants, which was quite common until 2006–2007, seems to have dropped drastically since then, according to veterinary services and local reports. Since 2011 and the official attachment of Mayotte to France, illegal transport by boat between Anjouan and Mayotte became also more controlled and thus more dangerous and expensive making it less interesting to transport cattle, though not impossible (couple of live ruminants are still seized every year). This aspect should still be carefully monitored to assess reemergence potential.

The impact of RVF in Mayotte (abortion or loss of production in cattle and human cases) is still unknown. Seroprevalence data showed the presence of IgG against RVF virus back to 2004 [7]. The circulation of RVF virus between 2004 and 2007 in Mayotte is unclear since only IgG but no IgM were found in cattle [7]. An increase in seroprevalence was observed between 2007 and 2009 with records of seroconversions in ruminants until 2011 [10]. Human cases were reported sporadically between 2007 and 2012 in the Comoros Archipelago [53,60]. Only one RVF related abortion in ruminant was identified in Mayotte during this period [61]. Only active surveillance would help assessing RVF real impact and whether the circulation has now faded out, like in 90% of our simulations five years after introduction, or has remained silent.

### Conclusion and modelling perspectives

Seasonality in vector abundance has been shown to play a key role in shaping RVF persistence through a complex interplay between biological characteristics of vectors and virus and host characteristics which importance varies from scenario to scenario. We have shown that RVF persistence may occur in a single tropical population with a low transmission scenario without the need for virus reintroduction and even with no or very low vertical transmission. Hence, active surveillance must be maintained to better understand the risk related to RVF persistence and to prevent any new introductions.

“This model can be easily adapted to other climatic conditions by rendering the emergence function relevant for other vector species and areas, or by using longitudinal relative vector abundance as observed in the field. In addition, parameters considered as constant in tropical areas (e.g. incubation rate) can be replaced by temperature functions, more relevant for temperate areas.”

As the heterogeneity in the spatial distribution of vectors and hosts may impact arbovirus spread to a large extent [62], an extension of our model to account for this heterogeneity (urban slums, forest, agricultural zones and the archipelago metapopulation situation) would enable us to better study RVF spread in a low transmission scenario and to link it with neighbouring territories dynamics. Human activities could also be included, through many aspects: (1) slaughter seasonality or farming practices may influence the persistence of RVF; (2) In urban areas, human behaviour might even be more influential than climate seasonality on vector dynamics [48,63]. Finally, building dynamic models for vector abundance adaptable to different localisations and environments have to be encouraged by extending our knowledge on vector biology from field and laboratory works.

## Supporting Information

S1 Fig Model code for Scilab. (PDF)

S2 Fig Average monthly precipitations and temperature observed in Mayotte. Precipitations were obtained from the agriculture department of Mayotte (DAAF) and temperature data were obtained from the public website of Meteofrance (https://donneespubliques.meteofrance.fr/).
(TXT)

S3 Fig Complete results of the sensitivity analysis on variance of model outputs. (PDF)
