# Supplementary material for: A Stochastic Model to Study Rift Valley Fever Persistence with Different Seasonal Patterns of Vector Abundance: New Insights on the Endemicity in the Tropical Island of Mayotte
Source: PLoS One. 2015 Jul 6;10(7):e0130838. doi: 10.1371/journal.pone.0130838 (PMC4493030; doi:10.1371/journal.pone.0130838)

Mean Rainfall 2004-2010 per month in different location in Mayotte

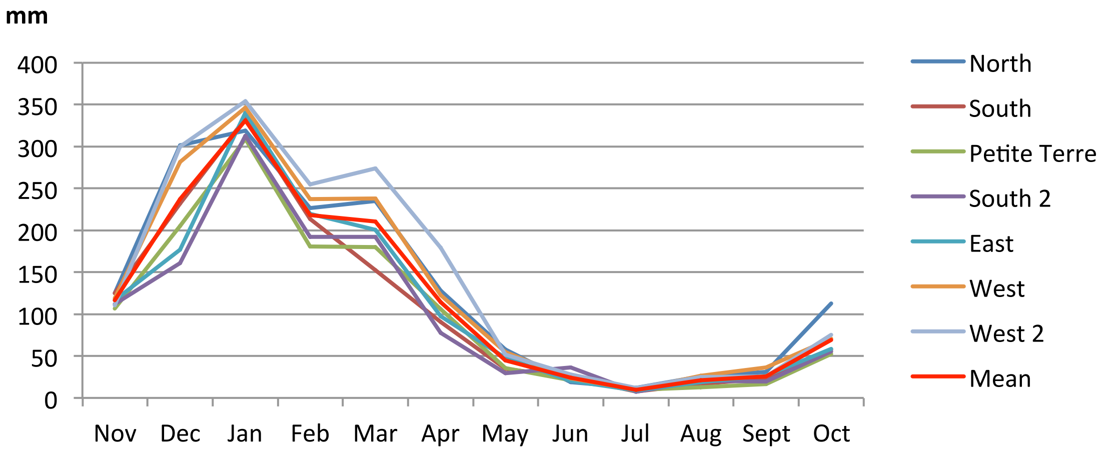

Location of the meteorological stations used in the graphics above :

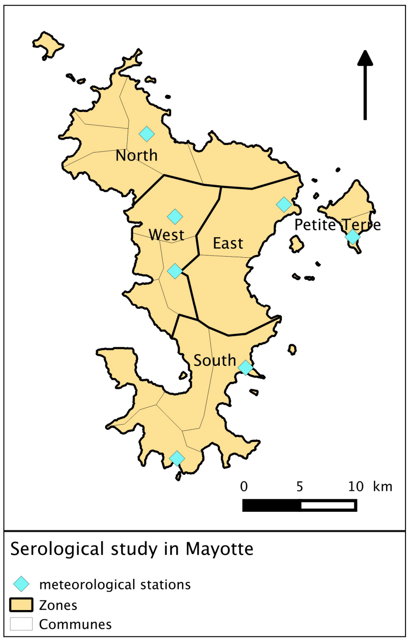

Mean Temperatures in Mayotte 2000-2009

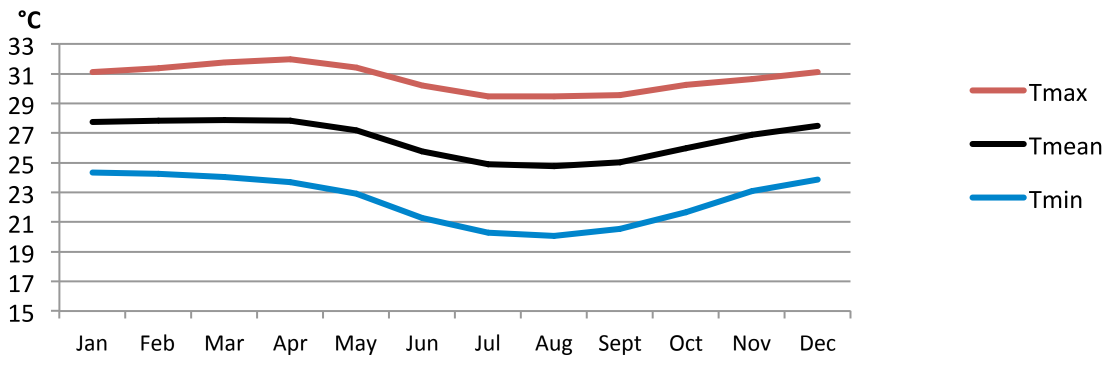

Supplement: S1 Fig — (PDF) [file pone.0130838.s001.pdf]
