# Supplementary material for: A Stochastic Model to Study Rift Valley Fever Persistence with Different Seasonal Patterns of Vector Abundance: New Insights on the Endemicity in the Tropical Island of Mayotte
Source: PLoS One. 2015 Jul 6;10(7):e0130838. doi: 10.1371/journal.pone.0130838 (PMC4493030; doi:10.1371/journal.pone.0130838)
Supplement: S3 Fig — (PDF) [file pone.0130838.s003.pdf]

		Persistence year 1				Persistence year 5				max I_{H1}				max I_{H2}				max I_{H3}				% direct infection				mean R_H				%mean IA J365->end			
		a	b	c	d	a	b	c	d	a	b	c	d	a	b	c	d	a	b	c	d	a	b	c	d	a	b	c	d	a	b	c	d
Host parameters	$b_H$et m_H	-	<2%	<2%	<2%	-	<2%	-	10%	<2%	<2%	<2%	<2%	-	5%	34%	60%	-	5%	39%	57%	5%	5%	4%	3%	<2%	<2%	9%	45%	15%	14%	10%	<2%
	c_{HH}	-	<2%	<2%	<2%	-	<2%	-	<2%	<2%	<2%	<2%	<2%	-	<2%	<2%	<2%	-	<2%	<2%	<2%	29%	34%	47%	43%	<2%	<2%	<2%	<2%	<2%	<2%	<2%	<2%
	$1/\delta_H$	-	<2%	<2%	<2%	-	<2%	-	<2%	3%	4%	4%	5%	-	<2%	<2%	<2%	-	<2%	<2%	<2%	<2%	<2%	<2%	<2%	<2%	<2%	<2%	<2%	<2%	<2%	<2%	<2%
	$1/\rho$	-	<2%	7%	43%	-	<2%	-	24%	45%	56%	67%	61%	-	<2%	13%	27%	-	2%	16%	26%	<2%	<2%	4%	3%	2%	3%	2%	<2%	13%	13%	14%	4%
	NH_{int}	-	9%	11%	5%	-	9%	-	<2%	5%	4%	2%	3%	-	3%	<2%	<2%	-	3%	<2%	<2%	6%	5%	3%	4%	7%	6%	4%	4%	<2%	<2%	<2%	3%
	c_{VH}	-	<2%	<2%	<2%	-	<2%	-	2%	5%	4%	3%	3%	-	3%	<2%	<2%	-	3%	<2%	<2%	6%	5%	4%	4%	7%	6%	4%	5%	<2%	<2%	<2%	3%
Vector parameters	b_V	-	<2%	<2%	4%	-	<2%	-	6%	<2%	<2%	<2%	<2%	-	<2%	<2%	<2%	-	<2%	<2%	<2%	<2%	<2%	<2%	<2%	<2%	<2%	<2%	<2%	<2%	<2%	<2%	5%
	m_V	-	15%	15%	21%	-	15%	-	4%	8%	4%	3%	4%	-	64%	44%	<2%	-	64%	34%	<2%	11%	10%	7%	6%	32%	42%	51%	11%	22%	24%	15%	52%
	q	-	19%	22%	9%	-	19%	-	17%	18%	15%	11%	12%	-	13%	<2%	4%	-	12%	<2%	5%	22%	21%	15%	17%	26%	22%	16%	19%	16%	16%	4%	<2%
	c_{HV}	-	10%	12%	5%	-	10%	-	6%	4%	3%	3%	3%	-	3%	<2%	<2%	-	3%	<2%	<2%	5%	5%	4%	4%	7%	6%	4%	5%	15%	15%	11%	<2%
	$1/\delta_V$	-	<2%	<2%	<2%	-	<2%	-	<2%	<2%	<2%	<2%	<2%	-	<2%	<2%	<2%	-	<2%	<2%	<2%	<2%	<2%	<2%	<2%	<2%	<2%	<2%	<2%	<2%	<2%	<2%	<2%
	α	-	<2%	<2%	<2%	-	<2%	-	5%	<2%	<2%	<2%	<2%	-	<2%	<2%	<2%	-	<2%	<2%	<2%	<2%	<2%	<2%	<2%	<2%	<2%	<2%	<2%	16%	14%	23%	8%
	ε	-	<2%	<2%	<2%	-	<2%		13%	<2%	<2%	<2%	<2%		<2%	<2%	<2%		<2%	<2%	<2%	<2%	<2%	<2%	<2%	<2%	<2%	<2%	<2%	<2%	<2%	7%	9%
	θ	-	9%	11%	<2%	-	9%	-	<2%	5%	4%	3%	4%	-	3%	<2%	<2%	-	3%	<2%	<2%	6%	5%	4%	5%	7%	6%	4%	3%	<2%	<2%	3%	<2%
	K_A	-	10%	12%	4%	-	10%	-	7%	5%	4%	3%	3%	-	3%	<2%	<2%	-	3%	<2%	<2%	6%	5%	4%	5%	7%	6%	4%	5%	<2%	<2%	<2%	3%
interactions	cvh:q	-	<2%	<2%	<2%	-	<2%	-	<2%	<2%	<2%	<2%	<2%	-	<2%	<2%	<2%	-	<2%	<2%	<2%	<2%	<2%	<2%	<2%	<2%	<2%	<2%	<2%	<2%	<2%	<2%	<2%
	mv:q	-	<2%	<2%	<2%	-	<2%	-	<2%	<2%	<2%	<2%	<2%	-	<2%	<2%	<2%	-	<2%	<2%	<2%	<2%	<2%	<2%	<2%	<2%	<2%	<2%	<2%	<2%	<2%	<2%	<2%
	chv:q	-	<2%	<2%	<2%	-	<2%	-	<2%	<2%	<2%	<2%	<2%	-	<2%	<2%	<2%	-	<2%	<2%	<2%	<2%	<2%	<2%	<2%	<2%	<2%	<2%	<2%	<2%	<2%	<2%	<2%
	q:KNA	-	<2%	<2%	<2%	-	<2%	-	<2%	<2%	<2%	<2%	<2%	-	<2%	<2%	<2%	-	<2%	<2%	<2%	<2%	<2%	<2%	<2%	<2%	<2%	<2%	<2%	<2%	<2%	<2%	<2%
	q:NHint	-	<2%	<2%	<2%	-	<2%	-	<2%	<2%	<2%	<2%	<2%	-	<2%	<2%	<2%	-	<2%	<2%	<2%	<2%	<2%	<2%	<2%	<2%	<2%	<2%	<2%	<2%	<2%	<2%	<2%
	q:Theta	-	<2%	<2%	<2%	-	<2%	-	<2%	<2%	<2%	<2%	<2%	-	<2%	<2%	<2%	-	<2%	<2%	<2%	<2%	<2%	<2%	<2%	<2%	<2%	<2%	<2%	<2%	<2%	<2%	<2%
contribution of parameters above 2%			72%	90%	90%		72%		93%	98%	98%	99%	98%		97%	91%	91%		99%	89%	89%	95%	95%	96%	95%	94%	96%	99%	97%	97%	96%	87%	88%
* not normally distributed		*				*		*						*				*															
